# Retraction Note: Integrative bioinformatics analysis of high-throughput sequencing and in vitro functional analysis leads to uncovering key hub genes in esophageal squamous cell carcinoma

**DOI:** 10.1186/s41065-026-00750-2

**Published:** 2026-10-10

**Authors:** Feng Shen, Xing Liu, Fengjiao Ding, Zhonglin Yu, Xinyi Shi, Lushan Cheng, Xuewei Zhang, Chengbao Jing, Zilong Zhao, Hongyou Cao, Bing Zhao, Jing Liu

**Affiliations:** 1 Clinical Laboratory, Ankang City Central Hospital, Ankang, 725000 China; 2 Oncology Department, Ankang City Central Hospital, Ankang, 725000 China; 3 Department of Thoracic Surgery, Ankang Central Hospital, Ankang, 725000 China; 4 Pathology Department, Ankang City Central Hospital, Ankang, 725000 China; 5 Oncology Department, People’s Hospital of Ankang City, Ankang, 725000 China

**Retraction Note: Hereditas 162, 38 (2025)**

**https://doi.org/10.1186/s41065-025-00398-4**

The Editor-in-Chief has retracted this article. After publication, concerns were raised regarding the in vitro images presented in Fig. 11 of this article, where the patterns of cells and colonies do not appear consistent with natural cell growth and proliferation. Additionally, the uncut western blot gel presented in the Supplementary Material appears to show contradictory results, with two proteins probed in adjacent lanes with different results. It has also been noted that a number of references in this article do not appear to support the statements preceding the citations.

The Editor-in-Chief therefore no longer has confidence in the presented data.

The publisher has not been able to obtain a current email address for authors Xinyi Shi and Zhonglin Yu. The other authors have not responded to correspondence regarding this retraction.

